# The SLC25A47 locus controls gluconeogenesis and energy expenditure

**DOI:** 10.1073/pnas.2216810120

**Published:** 2023-02-22

**Authors:** Jin-Seon Yook, Zachary H. Taxin, Bo Yuan, Satoshi Oikawa, Christopher Auger, Beste Mutlu, Pere Puigserver, Sheng Hui, Shingo Kajimura

**Affiliations:** ^a^Division of Endocrinology, Diabetes and Metabolism, Beth Israel Deaconess Medical Center, Harvard Medical School, Boston, MA 02115; ^b^Department of Molecular Metabolism, Harvard T. H. Chan School of Public Health, Boston, MA 02115; ^c^Department of Cell Biology, Harvard Medical School, Boston, MA 02115; ^d^Department of Cancer Biology, Dana-Farber Cancer Institute, Boston, MA 02115; ^e^HHMI, Chevy Chase, MD 20815

**Keywords:** bioenergetics, metabolism, obesity, type 2 diabetes, mitochondria

## Abstract

Mitochondria provide essential metabolites and adenosine triphosphate (ATP) for the regulation of energy homeostasis. For instance, liver mitochondria are a vital source of gluconeogenic precursors under a fasted state. However, the regulatory mechanisms at the level of mitochondrial membrane transport are not fully understood. Here, we report that a liver-specific mitochondrial inner-membrane carrier SLC25A47 is required for hepatic gluconeogenesis and energy homeostasis. Genome-wide association studies found significant associations between *SLC25A47* and fasting glucose, HbA1c, and cholesterol levels in humans. In mice, we demonstrated that liver-specific depletion of SLC25A47 impaired hepatic gluconeogenesis selectively from lactate, while significantly enhancing whole-body energy expenditure and the hepatic expression of FGF21. These metabolic changes were not a consequence of general liver dysfunction because acute SLC25A47 depletion in adult mice was sufficient to enhance hepatic FGF21 production, pyruvate tolerance, and insulin tolerance independent of liver damage and mitochondrial dysfunction. Mechanistically, SLC25A47 depletion leads to impaired hepatic pyruvate flux and malate accumulation in the mitochondria, thereby restricting hepatic gluconeogenesis. Together, the present study identified a crucial node in the liver mitochondria that regulates fasting-induced gluconeogenesis and energy homeostasis.

## Significance

Given the impenetrable nature of the mitochondrial inner-membrane, most of the known metabolite carrier proteins, including SLC25A family members, are ubiquitously expressed in mammalian tissues. One exception is SLC25A47, which is selectively expressed in the liver. The present study showed that depletion of SLC25A47 reduced mitochondrial pyruvate flux and hepatic gluconeogenesis under a fasted state, while activating energy expenditure. The present work offers a liver-specific target through which we can restrict hepatic gluconeogenesis, which is often in excess under hyperglycemic and diabetic conditions.

The role of mitochondria extends far beyond adenosine triphosphate (ATP) generation. Mitochondria serve as an essential organelle that supplies a variety of important metabolites to the cytosolic compartment and nucleus. An example is in the liver, wherein the mitochondria export phosphoenolpyruvate (PEP) and malate, which serve as gluconeogenic precursors in response to fasting. Under a fed condition, the mitochondria supply citrate that contributes to de novo lipogenesis (1, 2). In addition, mitochondrion-derived alpha-ketoglutarate (α-KG) functions as a cofactor of Jumonji C domain demethylases and ten-eleven translocation enzymes in the nucleus, thereby controlling the transcriptional program, a.k.a., retrograde signaling (3).

Mitochondrial flux in the liver is tightly regulated by hormonal cues, such as insulin and glucagon, and dysregulation of these processes profoundly impacts the maintenance of euglycemia, as often seen under the conditions of hyperglycemia and type 2 diabetes (4–6). For instance, elevated protein expression or activity of pyruvate carboxylase (PC), a mitochondrial matrix-localized enzyme that catalyzes the carboxylation of pyruvate to oxaloacetate (OAA), is associated with hyperglycemia (7, 8). On the other hand, liver-specific deletion of PC potently prevented hyperglycemia in diet-induced obese mice (9). Another example is the mitochondrion-localized phosphoenolpyruvate carboxykinase (PCK2, also known as M-PEPCK) that is expressed highly in the liver, pancreas, and kidney, where it catalyzes the conversion of OAA to PEP (10, 11). It has been demonstrated that activation of PCK2 in the liver enhanced the PEP cycle and potentiated gluconeogenesis (12, 13). In turn, depletion of PCK2 in the liver impaired lactate-derived gluconeogenesis and lowered plasma glucose, insulin, and triglycerides in mice (14). Accordingly, a better understanding of mitochondrial metabolite flux in the liver may provide insights into therapeutic strategies for the management of hyperglycemia and type 2 diabetes.

Of note, the mitochondrial inner-membrane is impermeable to metabolites relative to the outer membrane. As such, a variety of carrier proteins in the mitochondrial inner-membrane play essential roles in the regulation of metabolite transfer between the matrix and the cytosolic compartment (15, 16). As an example, mitochondrial pyruvate carrier (MPC) mediates the import of pyruvate into the matrix (17, 18). It has been demonstrated that liver-specific deletion of MPC1 or MPC2 reduced mitochondrial tricarboxylic acid (TCA) flux and impaired pyruvate-driven hepatic gluconeogenesis in diet-induced obese mice (19–21). Recent studies also reported the identification of SLC25A39, which is responsible for glutathione import (22), SLC25A44 for branched-chain amino acids import (23, 24), and SLC25A51 for nicotinamide adenine dinucleotide (NAD^+^) import (25–27).

**Fig. 1.**
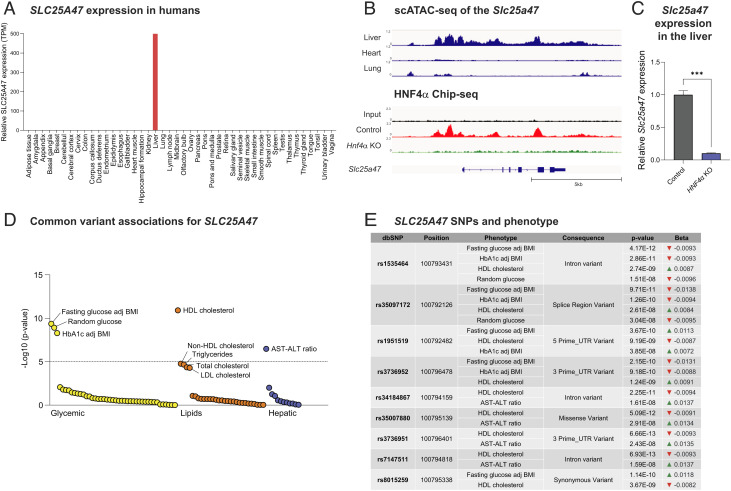
SLC25A47 is a liver-specific mitochondrial carrier that links to human metabolism. (*A*) Relative mRNA levels in indicated human tissues. The data obtained from Human Protein Atlas were analyzed. (*B*) *Upper*: The assay of transposase accessible chromatin sequencing (ATAC)-seq analysis of the *Slc25a47* gene locus in the liver, heart, and lung [data from gene expression omnibus (GEO) (GSE111586)]. *Lower*: The recruitment of HNF4α to the *Slc25a47* gene based on the chromatin immunoprecipitation sequencing (ChIP-seq) data of HNF4α [data from GEO (GSE90533)]. (*C*) The mRNA expression of liver *Slc25a47* in HNF4 null mouse embryos and controls (18.5-dpc). The data were obtained by analyzing the Affymetrix Mouse Genome 430 2.0 Array data (GSE3126). *n* = 3 for both groups. ***P < 0.001 by unpaired Student’s *t* test. (*D*) The phenome-wide association plot shows significant associations of *SLC25A47* for available traits generated by bottom-line metaanalysis across all datasets in the Common Metabolic Diseases Knowledge Portal. (*E*) The genetic associations between *SLC25A47* snips (SNPs) and indicated metabolic phenotypes.

Because of their essential roles, nearly all mitochondrial metabolite carriers (e.g., SLC25A family members) are ubiquitously expressed in mammalian tissues. However, there are two exceptions: uncoupling protein 1 (UCP1, also known as SLC25A7) that is selectively expressed in brown/beige fat (28), and an orphan carrier, SLC25A47, which is expressed selectively in the liver (Fig. 1*A*). SLC25A47 was previously described as a mitochondrial protein of which expression was down-regulated in hepatocellular carcinoma and that could reduce mitochondrial membrane potential in cultured Hep3B cells, a liver-derived epithelial cell line (29). In yeast, SLC25A47 overexpression elevated mitochondrial electron transport chain uncoupling, implicating its protective role against hepatic steatosis (30). In contrast, a recent study showed that genetic loss of *Slc25a47* led to mitochondrial dysfunction, mitochondrial stress, and liver fibrosis in mice (31). Given these apparently inconsistent reports, this study aims to determine the physiological role of SLC25A47 in systemic energy homeostasis.

## Results

### SLC25A47 Is a Liver-Specific Mitochondrial Carrier That Links to Human Metabolic Disease.

The SLC25A solute carrier proteins comprise 53 members in mammals, constituting the largest family of mitochondrial inter-membrane metabolite carriers (32). Among these 53 members, SLC25A47 is unique because this is the sole SLC25A member that is expressed selectively in the liver of mice (29, 31). We independently found that SLC25A47 is selectively expressed in the liver of humans (Fig. 1*A*) and in mice (*SI Appendix*, Fig. S1*A*). The publicly available single-cell RNA-seq dataset (33) shows that hepatocytes are the primary cell type that expresses *SLC25A47*, while Kupffer cells also express *SLC25A47* that account for approximately 10% of total transcripts in the liver (*SI Appendix*, Fig. S1*B*).

We next examined the genetic mechanism through which *Slc25a47* is selectively expressed in the liver. The analysis of assay of transposase accessible chromatin sequencing (ATAC-seq) data (GSE111586) found an open chromatin architecture in the *Slc25a47* gene locus (chromosome 12: 108,815,740 to 108,822,741) specific to the liver, whereas the same region appeared to form a heterochromatin structure in the heart and lung (Fig. 1 *B*, *Upper*). Notably, the euchromatin region of the *Slc25a47* gene contained binding sites of hepatocyte nuclear factor 4 alpha (HNF4α), to which HNF4α is recruited in the liver (Fig. 1 *B*, *Lower*). This result caught our attention because mutations of HNF4α are known to cause maturity-onset diabetes of the young 1, and it plays a central role in the regulation of hepatic and pancreatic transcriptional networks (34, 35). Importantly, HNF4α is required for the hepatic expression of *Slc25a47,* as the analysis of a previous microarray dataset (36) found that genetic loss of HNF4α significantly attenuated the expression of *Slc25a47* in the mouse liver (Fig. 1*C*).

Another important observation is in human genetic association studies from the Type 2 Diabetes Knowledge Portal (type2diabetesgenetics.org), wherein we found significant associations between *SLC25A47* and glycemic and lipid homeostasis. The notable associations include fasting glucose levels adjusted for body mass index (BMI), random glucose levels, HbA1c levels adjusted for BMI, high-density lipoprotein (HDL) cholesterol levels, and aspartate aminotransferase (AST)–alanine aminotransferase (ALT) ratio (Fig. 1*D*). One of the strongest single nucleotide polymorphisms (SNIPs) was located in the intronic region of *SLC25A47* (rs1535464), which showed significant associations with lower levels of fasting and random glucose, lower HbA1c levels adjusted for BMI, and higher HDL cholesterol levels (Fig. 1*E*). Similarly, another SNIP (rs35097172) in the regulatory region of *SLC25A47* was associated with lower levels of fasting/random glucose, HbA1c levels adjusted for BMI, and higher HDL cholesterol levels. These data indicate that SLC25A47 is involved in the regulation of glucose and lipid homeostasis, although how these snips (SNPs) affect *SLC25A47* expression remains unknown.

### Liver-Specific Depletion of SLC25A47 Protects against Body-Weight Gain and Lowers Plasma Cholesterol Levels.

**Fig. 2.**
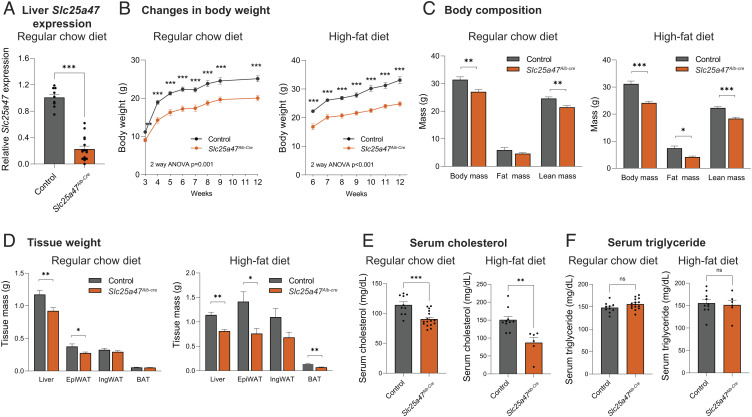
Metabolic characterization of liver-specific SLC25A47 deletion mice. (*A*) Relative liver *Slc25a47* mRNA levels in *Slc25a47^Alb-Cre^* (*n* = 10) and littermate controls (*n* = 6). (*B*) Changes in body weight of *Slc25a47^Alb-Cre^* mice and control on a regular chow diet and on a high-fat diet. Regular chow diet; *n* = 16 for *Slc25a47^Alb-Cre^*, *n* = 10 for controls. High-fat diet; *n* = 6 for *Slc25a47^Alb-Cre^*, *n* = 11 for controls. *P*-value determined by two-way ANOVA followed by unpaired Student’s *t* test. (*C*) Body composition of mice at 16 wk of regular chow diet and at 6 wk of high-fat diet. Regular chow diet; *n* = 11 for *Slc25a47^Alb-Cre^*, *n* = 10 for controls. High-fat diet; *n* = 6 for *Slc25a47^Alb-Cre^*, *n* = 11 for controls. (*D*) Indicated tissue weight of mice in (*B*). (*E*) Serum cholesterol levels of mice at 12 wk of age on a regular chow diet and after 6 wk of high-fat diet. Regular chow diet; *n* = 16 for *Slc25a47^Alb-Cre^*, *n* = 10 for controls. High-fat diet; *n* = 6 for *Slc25a47^Alb-Cre^*, *n* = 10 for controls. (*F*) Serum TG levels of mice in (*E*). ns, not significant. *A*–*E*, *P < 0.05, **P < 0.01, ***P < 0.001 by unpaired Student’s *t* test.

To determine the physiological role of SLC25A47 in energy homeostasis, we next developed mice that lacked SLC25A47 in a liver-specific manner by crossing *Slc25a47*^flox/flox^ mice with Albumin-Cre (*Alb*-Cre; *Slc25a47*^flox/flox^, herein *Slc25a47^Alb-Cre^* mice). We validated that the liver of *Slc25a47^Alb-Cre^* mice expressed significantly lower levels of *Slc25A47* messenger RNA (mRNA) than littermate control mice (*Slc25a47*^flox/flox^) by 80 % (Fig. 2*A*). The remaining mRNA in *Slc25a47^Alb-Cre^* mice could be attributed to inefficient Cre expression or the transcripts in nonhepatocytes, such as Kupffer cells. The expression of the *Slc25a47* neighboring genes, including *Wdr25, Begain, Dlk1, Meg3, Slc25a29, Yy1,* and *Degs2*, was not altered in the liver of *Slc25a47^Alb-Cre^* mice relative to control mice (*SI Appendix*, Fig. S2*A*).

At birth, there was no difference in the body weight and body size between *Slc25a47^Alb-Cre^* mice and littermate control mice (*SI Appendix*, Fig. S2*B*). However, *Slc25a47^Alb-Cre^* mice gained significantly less weight than controls at 3 wk of age and thereafter on a regular-chow diet (Fig. 2 *B*, *Left*). This phenotype was more profound when mice at 6 wk of age were fed on a high-fat diet (HFD, 60% fat) (Fig. 2 *B*, *Right*). The difference in body weight arose from reduced adipose tissue mass and lean mass both on a regular-chow diet and a high-fat diet (Fig. 2*C*). At tissue levels, adipose tissue and liver mass were lower in *Slc25a47^Alb-Cre^* mice relative to control mice (Fig. 2*D*).

Additionally, we found significantly lower serum levels of total cholesterol in *Slc25a47^Alb-Cre^* mice than those in controls both on regular-chow and high-fat diets (Fig. 2*E*). On the other hand, we observed no difference in serum triglyceride (TG) levels between the two groups both on regular-chow and high-fat diets (Fig. 2*F*). We found no difference in serum ALT, AST, and albumin levels on a high-fat diet, although serum ALT and AST levels were higher in *Slc25a47^Alb-Cre^* mice at 12 wk of age on a regular-chow diet (*SI Appendix*, Fig. S2 *C–E*).

### Depletion of SLC25A47 Led to Elevated Whole-Body Energy Expenditure.

**Fig. 3.**
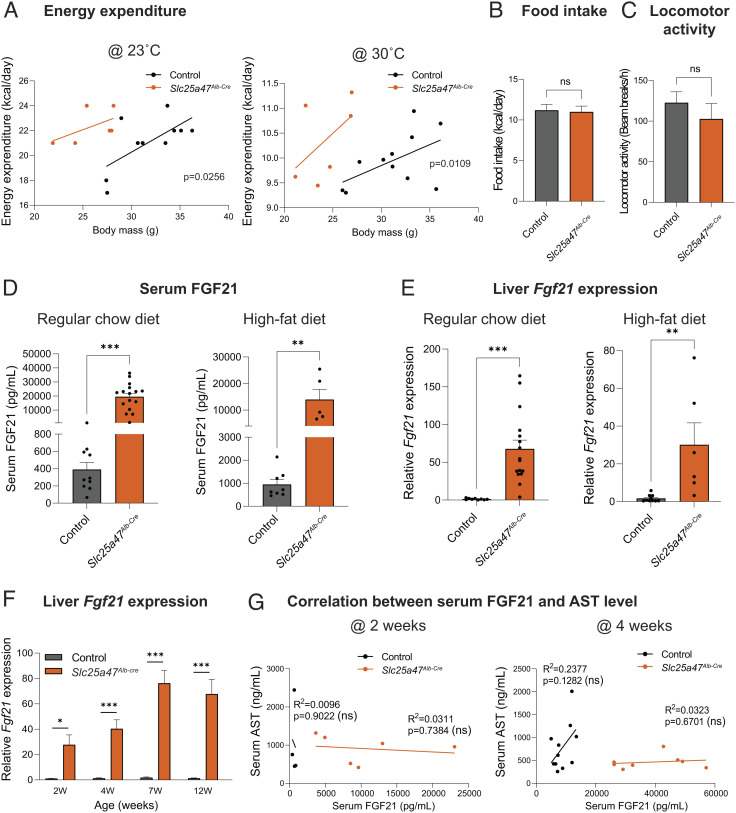
Depletion of SLC25A47 elevated whole-body energy expenditure. (*A*) CaIR-analysis of covariance (ANCOVA) analysis of *Slc25a47^Alb-Cre^* mice and littermate controls at 23 °C after 1 wk on a high-fat diet and at 30 °C after 3 wk on a high-fat diet. *n* = 6 for *Slc25a47^Alb-Cre^*, *n* = 11 for controls. (*B*) Food intake of mice in (*A*). (*C*) Locomotor activity of mice in (*A*). (*D*) Serum FGF21 levels of mice at 12 wk of age on a regular chow diet and a high-fat diet for 6 w. Regular chow diet; *n* = 16 for *Slc25a47^Alb-Cre^*, *n* = 10 for controls. High-fat diet; *n* = 16 for *Slc25a47^Alb-Cre^*, *n* = 10 for controls. (*E*) Relative hepatic *Fgf21* expression of mice in (*D*). (*F*) Relative hepatic *Fgf21* expression of mice on a regular chow diet at indicated ages. *n* = 6 for *Slc25a47^Alb-Cre^*, *n* = 4 for controls (2 wk), *n* = 8 for *Slc25a47^Alb-Cre^*, *n* = 14 for controls (4 wk), *n* = 14 for *Slc25a47^Alb-Cre^*, *n* = 14 for controls (7 wk), *n* = 16 for *Slc25a47^Alb-Cre^*, *n* = 9 for controls (12 wk). (*G*) Correlation between serum FGF21 levels and serum AST levels of mice at 2 wk (*Left*) and 4 wk of age (*Right*) on a regular chow diet. *n* = 6 for *Slc25a47^Alb-Cre^*, *n* = 4 for controls (2 wk), *n* = 8 for *Slc25a47^Alb-Cre^*, *n* = 11 for controls (4 wk). ns, not significant, by simple linear regression. *B*–*F*, ns, not significant, *P < 0.05, **P < 0.01, ***P < 0.001 by unpaired Student’s *t* test.

Given the difference in body weight between *Slc25a47^Alb-Cre^* mice and control mice, we examined the whole-body energy expenditure using metabolic cages. Regression-based analysis of energy expenditure by CaIR-analysis of covariance (ANCOVA) (37) showed that *Slc25a47^Alb-Cre^* mice exhibited significantly higher whole-body energy expenditure (kcal/day) independent of body mass at 23 °C. The difference remained significant when mice were kept at 30 °C (Fig. 3*A*). On the other hand, there was no difference in their food intake and locomotor activity between the genotypes (Fig. 3 *B* and *C*).

A possible explanation for the high energy expenditure might be the enhanced thermogenic capacity of brown adipose tissue (BAT) or its sensitivity to β3-adrenergic receptor (β3-AR) signaling. Accordingly, we tested the hypothesis by examining BAT thermogenesis in response to a β3-AR agonist (CL316,243) at 30 °C. This is a gold-standard method to determine BAT thermogenic responses to β3-AR stimuli, while excluding the contribution of shivering thermogenesis by skeletal muscle (38). We found that a single administration of β3-AR agonist (CL316,243) at 0.5 mg/kg (high dose) potently increased whole-body energy expenditure both in *Slc25a47^Alb-Cre^* and littermate controls to a similar degree (*SI Appendix*, Fig. S3*A*). This result suggests that the cell-intrinsic thermogenic capacity of BAT, if maximumly activated by a β3-AR stimulus, appears comparable between the two groups. Accordingly, we asked if there was any change in circulating hormonal factors that influenced whole-body energy expenditure of *Slc25a47^Alb-Cre^* mice. In this regard, FGF21 is a probable candidate because it is a well-established endocrine hormone that increases energy expenditure by activating the sympathetic nervous system (39). Consistent with the recent work (31), we found that serum levels of FGF21 in *Slc25a47^Alb-Cre^* mice were significantly higher relative to littermate controls both on regular-chow and high-fat diets (Fig. 3*D*). The increase in circulating FGF21 levels was due to elevated *Fgf21* transcription in the liver (Fig. 3*E*). This is in agreement with the previous work demonstrating that the liver is the primary source of circulating FGF21 (40).

Of note, elevated *Fgf21* gene expression in *Slc25a47^Alb-Cre^* mice was already observed at 2 wk of age, a time point in which there was no difference in body weight, serum ATL/AST levels, and mitochondrial stress-related genes in the liver (Fig. 3*F* and *SI Appendix*, Fig. S3 *B–D*). Importantly, there was no correlation between serum FGF21 levels and AST levels in control and *Slc25a47^Alb-Cre^* mice at 2 and 4 wk of age (Fig. 3*G*). The results indicate that the stimulatory effect of SLC25A47 loss on FGF21 expression is not merely a consequence of liver damage. We addressed this point further in the following sections.

### SLC25A47 Is Required for Pyruvate-Derived Hepatic Gluconeogenesis In Vivo.

**Fig. 4.**
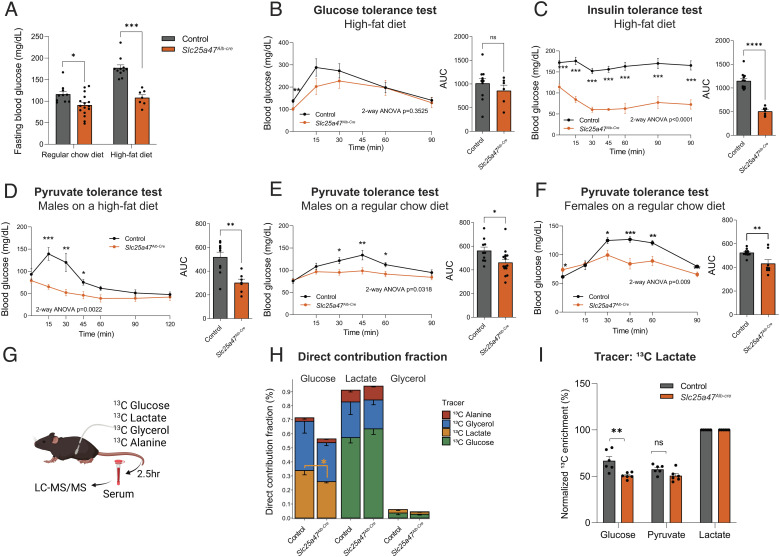
SLC25A47 is required for pyruvate-derived hepatic gluconeogenesis. (*A*) Fasting blood glucose levels (6 h) in *Slc25a47^Alb-Cre^* mice and littermate controls at 7 wk of age on a regular chow diet and at 4 wk on a high-fat diet. Regular diet; *n* = 16 for *Slc25a47^Alb-Cre^*, *n* = 10 for controls. High-fat diet; *n* = 6 for *Slc25a47^Alb-Cre^*, *n* = 11 for controls. *P < 0.05, ***P < 0.001 by unpaired Student’s t test. (*B*) Glucose tolerance test in *Slc25a47^Alb-Cre^* mice (*n* = 6) and littermate controls (*n* = 11) at 4 wk of high-fat diet. 6-h-fasted mice received glucose (2 g kg^−1^ body weight, *i.p.*). *Right*: Area under the curve (AUC) of the data. ns, not significant. (*C*) Insulin tolerance test in *Slc25a47^Alb-Cre^* mice (*n* = 7) and littermate controls (*n* = 11) at 4 wk of high-fat diet. 6-h-fasted mice received insulin (0.4 U kg^−1^ body weight, *i.p.*). (*D*) Pyruvate tolerance test in *Slc25a47^Alb-Cre^* mice (*n* = 6) and littermate controls (*n* = 11) on a high-fat diet for 3 wk. 16-h-fasted mice received pyruvate (2 g kg^−1^ body weight, *i.p.*). (*E*) Pyruvate tolerance test in male *Slc25a47^Alb-Cre^* mice (*n* = 16) and littermate controls (*n* = 10) on a regular chow diet. (*F*) Pyruvate tolerance test in female *Slc25a47^Alb-Cre^* mice (*n* = 8) and littermate controls (*n* = 12) on a regular chow diet. (*G*) Schematic illustration of tracer experiments. Fasted mice on a regular chow diet were infused with indicated ^13^C-labeled tracers via the catheter. (*H*) Direct contribution of ^13^C-labeled tracers to glucose, lactate, and glycerol in (*G*). *n* = 6 for *Slc25a47^Alb-Cre^*, *n* = 6 for controls. *P < 0.05 by unpaired Student’s *t* test. (*I*) The relative contribution of ^13^C-labeled lactate to circulating levels of glucose, pyruvate, and lactate in (*G*). **P < 0.01. *B*–*F*, *P*-value determined by two-way ANOVA followed by Fisher's least significant difference (LSD) test. AUC: *P < 0.05, **P < 0.01, ****P < 0.0001 by unpaired Student’s *t* test.

We next examined the extent to which SLC25A47 regulates systemic glucose homeostasis. This is based on the observation that fasting glucose levels of *Slc25a47^Alb-Cre^* mice were consistently lower than littermate controls both on regular-chow and high-fat diets (Fig. 4*A*). At 4 wk of high-fat diet, we found no major difference in glucose tolerance between the two groups, although fasting glucose levels were lower in *Slc25a47^Alb-Cre^* mice than control mice (Fig. 4*B*). In contrast, *Slc25a47^Alb-Cre^* mice exhibited significantly higher insulin tolerance than controls in response to insulin at a low dose (0.4 U/kg) (Fig. 4*C*). It is notable that *Slc25a47^Alb-Cre^* mice remained hypoglycemic (<70 mg/dL) following insulin administration.

Pyruvate tolerance tests found that *Slc25a47^Alb-Cre^* mice at 3 wk of high-fat diet exhibited significantly lower hepatic gluconeogenesis than control mice (Fig. 4*D*). Of note, the difference in pyruvate tolerance was independent of diet and sex, as we observed consistent results both in male and female mice on a regular-chow diet (Fig. 4 *E* and *F*). On the other hand, there was no difference in glucose-stimulated serum insulin levels and hepatic glycogen contents between the two groups (*SI Appendix*, Fig. S4 *A* and *B*). These results led to the hypothesis that the lower fasting glucose levels seen in *Slc25a47^Alb-Cre^* mice are attributed to reduced hepatic gluconeogenesis, rather than impaired glycogenolysis or elevated insulin sensitivity in the skeletal muscle.

To test the hypothesis, we next examined the contribution of hepatic gluconeogenesis to circulating glucose by infusing fasted mice with U-^13^C-labeled lactate or ^13^C-labeled glucose. To examine the relative contribution of other gluconeogenic precursors to blood glucose, we also infused fasted mice with U-^13^C-labeled glycerol and U-^13^C-alanine (Fig. 4*G*). During the infusion, we collected and analyzed serum from fasted mice using liquid-chromatography–mass spectrometry (LC–MS), as described in recent studies (41, 42). We used ^13^C-lactate as a gluconeogenic precursor instead of pyruvate because circulating lactate is the primary contributor to gluconeogenesis and in rapid exchange with pyruvate (43).

The analyses showed that glucose production from ^13^C-lactate was significantly lower in *Slc25a47^Alb-Cre^* mice than in control mice (Fig. 4*H* orange bars). Notably, this impairment was selective to the lactate-to-glucose conversion, as we found no significant difference in glucose production from ^13^C-glycerol between the two groups (Fig. 4*H* blue bars and *SI Appendix*, Fig. S4*C*). The relative contribution of alanine to serum glucose was far less than lactate, with no statistical difference between the genotypes (Fig. 4*H* red bars). The lactate-to-pyruvate conversion was unaffected in *Slc25a47^Alb-Cre^* mice, suggesting that impaired gluconeogenesis from lactate is attributed to reduced pyruvate utilization in the liver (Fig. 4*I*). We also found no difference in the conversion from ^13^C-glucose to pyruvate and lactate (*SI Appendix*, Fig. S4*D*). These results indicate that SLC25A47 is required selectively for gluconeogenesis from lactate under a fasted condition, whereas it is dispensable for gluconeogenesis from other substrates.

### Acute Depletion of SLC25A47 Improved Glucose Homeostasis without Causing Liver Damage.

**Fig. 5.**
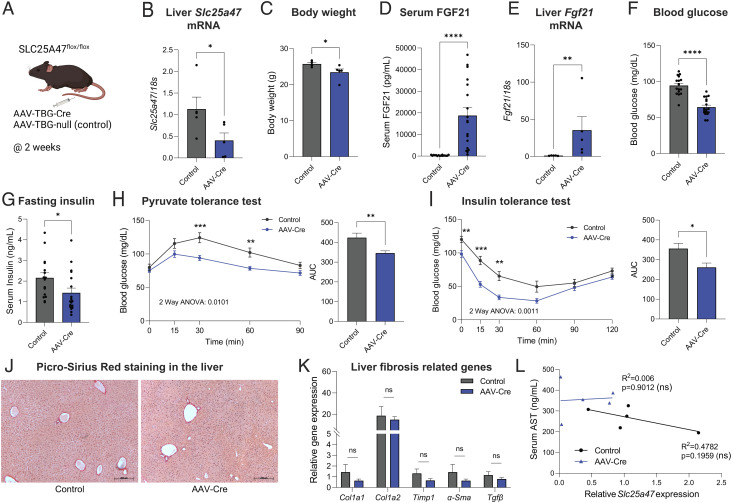
Acute depletion of SLC25A47 improves glucose homeostasis independent of liver damage. (*A*) Schematic illustration of acute depletion of *Slc25a47* study. *Slc25a47*^flox/flox^ mice at 7 wk of age on a regular chow diet received AAV-Cre or AAV-null (control) via tail-vein. (*B*) Relative hepatic *Slc25a47* mRNA levels of mice at 2 wk after AAV injection. *n* = 5 for both groups. *P < 0.05 by Mann–Whitney *U* test. (*C*) Body weight of mice in (*B*). *P < 0.05 by unpaired Student’s *t* test. (*D*) Serum FGF21 levels of mice after 2 wk of AAV injection. *n* = 16 for controls, *n* = 18 for AAV-Cre. ****P < 0.0001 by unpaired Student’s *t* test. (*E*) Relative expression of hepatic *Fgf21* of mice in (*B*). **P < 0.01 by Mann–Whitney *U* test. (*F*) Fasting blood glucose levels (6 h) of mice in (*B*). ****P < 0.0001 by unpaired Student’s *t* test. (*G*) Fasting insulin levels of mice in (*A*). *n* = 16 for controls, *n* = 18 for AAV-Cre. *P < 0.05 by unpaired Student’s *t* test. (*H*) Pyruvate tolerance test at 2 wk after AAV injection. Fasted mice received pyruvate (2 g kg^−1^ body weight, *i.p.*). *n* = 15 for controls, *n* = 16 for AAV-Cre. *P*-value determined by two-way ANOVA followed by Fisher's LSD test. *Right*: AUC. **P < 0.01 by unpaired Student’s *t* test. (*I*) Insulin tolerance test in *Slc25a47*^flox/flox^ mice at 6 wk. Fasted mice received insulin (0.4 U kg^−1^ body weight, *i.p.*). *n* = 11 for controls, *n* = 13 for AAV-Cre. *Right*: AUC. *P < 0.05 by unpaired Student’s *t* test. (*J*) Representative liver Picro-Sirius Red staining of mice in (*B*). Scale = 200 μm. (*K*) Relative liver expression of fibrosis marker genes of mice in (*B*). ns, not significant (*L*) Correlation between serum AST levels and hepatic *Slc25a47* expression of mice in (*B*).

A recent work suggested the possibility that the metabolic changes in *Slc25a47^Alb-Cre^* mice, such as elevated FGF21 and impaired glucose production, were merely secondary to general hepatic dysfunction and fibrosis (31). To exclude metabolic complications caused by chronic deletion of SLC25A47, particularly during the prenatal and early postnatal periods, we aimed to acutely deplete SLC25A47 in adult mice. To this end, we acutely depleted SLC25A47 in adult mice by delivering adeno-associated virus (AAV)-thyroxine binding globulin (TBG)-Cre or AAV-TBG-null (control) into the liver of *Slc25a47*^flox/flox^ mice via tail-vein (Fig. 5*A*). AAV-Cre administration successfully reduced *Slc25a47* mRNA expression by approximately 50% (Fig. 5*B*). Although the depletion efficacy of AAV-Cre was less than the genetic approach using *Albumin*-Cre, this model gave us an opportunity to determine the extent to which acute and partial depletion of SLC25A47 in adult mice sufficiently affect hepatic glucose production and energy expenditure, while avoiding metabolic complications associated with chronic SLC25A47 deletion.

After 2 wk of AAV administration, we found that acute SLC25A47 depletion led to reduced body-weight gain (Fig. 5*C* and *SI Appendix*, Fig. S5*A*) and increased serum FGF21 levels (Fig. 5*D*). The increase in serum FGF21 levels was associated with elevated hepatic FGF21 mRNA expression (Fig. 5*E*). Consistent with the observations in *Slc25a47^Alb-Cre^* mice, acute SLC25A47 depletion resulted in reduced fasting serum glucose levels (Fig. 5*F*) and insulin levels (Fig. 5*G*). Importantly, acute SLC25A47 depletion improved systemic pyruvate tolerance (Fig. 5*H*) and insulin tolerance (Fig. 5*I*). In contrast, acute SLC25A47 did not alter systemic glycerol tolerance, although there was a modest change at later time points after glycerol administration (*SI Appendix*, Fig. S5*B*). The difference in glycerol tolerance at later time points is likely because glycerol-derived glucose is converted to lactate in peripheral tissues, which is eventually utilized as a gluconeogenic substrate (44).

Next, we examined whether such metabolic changes were associated with liver injury in vivo. Histological analyses by Picro-Sirius Red staining did not find any noticeable sign of liver fibrosis (Fig. 5*J*). Similarly, histological analyses by hematoxylin and eosin (H&E) staining found no difference between control vs. AAV-Cre injected mice (*SI Appendix*, Fig. S5*C*). Furthermore, acute SLC25A47 depletion did not alter the expression of liver fibrosis marker genes (Fig. 5*K*). Also, we found no significant correlation between serum AST levels and hepatic SLC25A47 expression (Fig. 5*L*) and between serum AST levels and FGF21 levels (*SI Appendix*, Fig. S5*D*). Moreover, we observed no significant difference in the Complex I and II activities of isolated liver mitochondria between the two groups (*SI Appendix*, Fig. S5*E*). These data suggest that acute SLC25A47 depletion sufficiently enhanced hepatic FGF21 expression, pyruvate tolerance, and insulin tolerance independent of liver damage and hepatic mitochondrial dysfunction.

### SLC25A47 Is Required for Mitochondrial Pyruvate Flux and Malate Export.

**Fig. 6.**
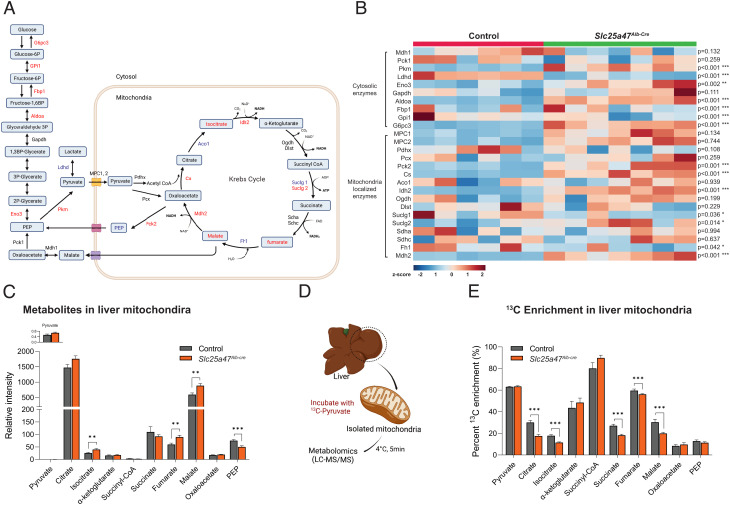
SLC25A47 is required for mitochondrial pyruvate efflux and malate export. (*A*) Summary of liver RNA-seq and mitochondrial metabolomics data in *Slc25a47^Alb-Cre^* mice and littermate controls on a regular chow diet. Red letters: up-regulated in *Slc25a47^Alb-Cre^* relative to controls. Blue letters: down-regulated in *Slc25a47^Alb-Cre^*. Black letters: no statistical changes. (*B*) Heatmap displaying liver mRNA levels of cytosolic gluconeogenic enzymes and mitochondria localized enzymes in fasted 12-wk-old *Slc25a47^Alb-Cre^* mice (*n* = 7) and controls (*n* = 6). Data transcript per million (TPM) are expressed as z-scores; blue (low expression), red (high expression). *P*-value determined by unpaired Student’s *t* test. (*C*) Relative metabolite levels in the liver mitochondria of fasted *Slc25a47^Alb-Cre^* mice (*n* = 14) and controls (*n* = 14). Data were normalized to mitochondrial protein levels (ug/mL). *P*-value determined by unpaired Student’s *t* test. (*D*) Schematic illustration of tracer experiments in isolated mitochondria. (*E*) The relative contribution of ^13^C-labeled pyruvate to indicated metabolites in the mitochondria. *n* = 14 for *Slc25a47^Alb-Cre^*, *n* = 14 for lcontrols. Data were normalized to mitochondrial protein. *P*-value determined by unpaired Student’s *t* test.

We next asked which steps of the lactate-derived hepatic gluconeogenesis were altered in *Slc25a47^Alb-Cre^* mice. To this end, we took unbiased omics approaches—RNA-seq and mitochondrial metabolomics analyses—in the liver of *Slc25a47^Alb-Cre^* mice and littermate controls under a fasted condition. The summary of the results is shown in Fig. 6*A*. The RNA-seq data analysis found that the liver of *Slc25a47^Alb-Cre^* mice expressed significantly higher levels of *Pkm*, *Eno3, Aldoa*, *Fbp1*, *Gpi1*, and *G6pc3* (Fig. 6*B*), suggesting a compensatory upregulation of gluconeogenic gene expression in *Slc25a47^Alb-Cre^* mice.

A notable finding is the distinct regulation of mitochondrial matrix-localized enzymes vs. cytosolic enzymes: We found that the expression of the mitochondrial TCA cycle enzymes, such as citrate synthase (*Cs*), the mitochondrial form of isocitrate dehydrogenase (*Idh2*), and *Suclg2* (the subunits of succinate-CoA ligase) was significantly up-regulated in the liver of *Slc25a47^Alb-Cre^* mice relative to controls. In addition, the expression of *Pck2*, the M-PEPCK that converts OAA to PEP within the mitochondria, was up-regulated in the liver of *Slc25a47^Alb-Cre^* mice. In contrast, the expression of the cytosolic form of PEPCK (*Pck1*) was unchanged. Similarly, the expression of *Mdh2*, which catalyzes the conversion between OAA and malate in the mitochondria, was significantly elevated in the liver of *Slc25a47^Alb-Cre^* mice, whereas the expression of *Mdh1*, the cytosolic form, showed a trend of down-regulation. These results suggest that SLC25A47 loss leads to a distinct gene expression pattern of mitochondrial vs. cytosolic enzymes that control hepatic gluconeogenesis.

The mitochondrial metabolomics analysis revealed that the liver mitochondria of *Slc25a47^Alb-Cre^* mice accumulated significantly higher levels of isocitrate, fumarate, and malate than those of control mice (Fig. 6*C*). In contrast, mitochondrial PEP contents were lower in *Slc25a47^Alb-Cre^* livers relative to controls. We found no difference in the mitochondrial contents of pyruvate, citrate, α-KG, succinyl CoA, succinate, and OAA between the two groups. Additionally, there was no difference in the mitochondrial contents of cofactors required for the TCA cycle reactions, such as coenzyme A, reduced nicotinamide adenine dinucleotide (NADH), nicotinamide adenine dinucleotide phosphate (NADP^+^), NADPH, and flavin adenine dinucleotide (FAD), although mitochondrial NAD^+^ and guanosine triphosphate (GTP) levels were higher in *Slc25a47^Alb-Cre^* mice than controls (*SI Appendix*, Fig. S6*A*).

The above data led to the hypothesis that SLC25A47 controls either pyruvate import to the mitochondrial matrix or pyruvate flux within the mitochondria. To test this, we isolated mitochondria from the liver of *Slc25a47^Alb-Cre^* mice and littermate controls under a fasted condition. The isolated mitochondria were incubated with [U-^13^C] labeled pyruvate and subsequently analyzed by LC–MS/MS (Fig. 6*D*). We found no difference in the mitochondrial contents of ^13^C-pyruvate levels between the two groups, suggesting that mitochondrial pyruvate uptake per se was not altered in the liver of *Slc25a47^Alb-Cre^* mice (Fig. 6*E*). This is in agreement with the data that the expression of MPC1 and MPC2 was not different between the genotypes (Fig. 6*B*). On the other hand, the enrichments of ^13^C-labeled citrate, isocitrate, succinate, fumarate, and malate were significantly lower in the mitochondria of *Slc25a47^Alb-Cre^* mice than those in controls (Fig. 6*E*). There was no difference in ^13^C-labeled OAA and PEP between the groups. Together, these results suggest that genetic loss of SLC25A47 impaired mitochondrial pyruvate flux, leading to an accumulation of fumarate, malate, and isocitrate in the liver mitochondria. Impaired export of malate from the mitochondria into the cytosolic compartment leads to reduced lactate-derived hepatic gluconeogenesis under a fasted condition.

## Discussion

Mitochondrial flux in the liver is highly nutrition-dependent. Under a fed condition, malate is imported into the mitochondrial matrix in exchange for α-KG via mitochondrial α-KG/malate carrier (SLC25A11) as a part of the malate-aspartate shuttle, a mechanism to transport reducing equivalents (NADH) into the mitochondrial matrix (45). In addition, mitochondrial dicarboxylate carrier SLC25A10 can mediate the import of malate into the mitochondrial matrix in addition to malonate, succinate, phosphate, sulfate, and thiosulfate (46). Under a fasted state, when liver glycogen is depleted, malate is exported from the mitochondrial matrix into the cytosolic compartment, where it is converted to OAA by MDH1 and utilized as a gluconeogenic substrate. However, what controls the nutrition-dependent mitochondrial malate flux remains elusive. The present work showed that SLC25A47 depletion led to an accumulation of mitochondrial malate and reduced hepatic gluconeogenesis, without affecting gluconeogenesis from glycerol. The results indicate that SLC25A47 mediates the export of mitochondrion-derived malate into the cytosol. However, the present study could not exclude the possibility that SLC25A47 mediates the transport of cofactors needed for mitochondrial pyruvate flux, although we found no difference in the mitochondrial contents of coenzyme A and NADH between the genotypes. Our future study aims to determine the specific substrate of SLC25A47 by biochemically reconstituting this protein in a cell-free system, such as liposomes.

The present work showed that depletion of SLC25A47 reduced mitochondrial pyruvate flux, thereby restricting lactate-derived hepatic gluconeogenesis and preventing hyperglycemia. This is in alignment with several mouse models with impaired mitochondrial pyruvate flux in the liver. For instance, liver-specific depletion of pyruvate carboxylase (PC limits the supply of pyruvate-derived OAA in the mitochondria, leading to reduced TCA flux and hepatic gluconeogenesis (9). Similarly, liver-specific depletion of the MPC1 or MPC2 or the M-PEPCK reduces hepatic gluconeogenesis and protects mice against diet-induced hyperglycemia (14, 19–21). A recently developed noninvasive method, positional isotopomer NMR tracer analysis, would be instrumental to determine how SLC25A47 loss alters the rates of hepatic mitochondrial citrate synthase flux vs. PC flux (35).

It is worth pointing out that elevated energy expenditure and reduced body weight are unique to *Slc25a47^Alb-Cre^* mice. Indeed, no changes in energy expenditure and body weight were seen in mice that lacked MPC1/2 or M-PEPCK relative to the respective controls. Elevated energy expenditure of *Slc25a47^Alb-Cre^* mice appears to be attributed to elevated FGF21 as recent work demonstrated that deletion of FGF21 abrogated the effects of SLC25A47 on energy expenditure and body weight (31). Importantly, our results suggest that partial SLC25A47 depletion was sufficient to stimulate FGF21 production independently from liver damage. It is conceivable that changes in mitochondrion-derived metabolites, such as malate and others, control the transcription of FGF21 via retrograde signaling (3). Our future study will explore the mechanisms through which SLC25A47-mediated mitochondrial signals control the nuclear-coded transcriptional program in a nutrition-dependent manner. In addition, genetic rescue experiments, such as ectopically reintroducing SLC25A47 into the liver of *Slc25a47^Alb-Cre^* mice will determine the direct vs. indirect actions of SLC25A47 on gluconeogenesis and energy expenditure.

With these results in mind, we consider that SLC25A47 is a plausible target for hyperglycemia and type 2 diabetes for the following reasons. First, excess hepatic gluconeogenesis is commonly seen in human hyperglycemia and type 2 diabetes (4–6). Notably, genome-wide association studies (GWAS) data found significant associations between *SLC25A47* and glycemic homeostasis in humans—particularly, several SNPs in the *SLC25A47* were significantly associated with lower levels of glucose and HbA1c adjusted for BMI, although how these SNPs affect *SLC25A47* expression awaits future studies. Second, SLC25A47 is exceptionally unique among 53 members of the mitochondrial SLC25A carriers, given its selective expression in the liver. This tissue specificity makes SLC25A47 an attractive therapeutic target, considering the recent successful examples in which liver-targeting mitochondrial uncouplers protected mice against type 1 and type 2 diabetes, hepatic steatosis, and cardiovascular complications (47–49). A potential caveat is the detrimental effect associated with chronic SLC25A47 deletion, such as mitochondrial stress, lipid accumulation, and fibrosis (31). However, our data showed that acute depletion of SLC25A47 by ~50% sufficiently restricted gluconeogenesis and enhanced insulin tolerance in adult mice without causing liver fibrosis and mitochondrial dysfunction. Thus, it is conceivable that temporal and partial inhibition of SLC25A47 using small-molecule inhibitors or antisense oligos would be effective in restricting excess hepatic gluconeogenesis while avoiding the detrimental side effects.

## Method

### Animal Study.

All the animal experiments in this study were performed in compliance with protocols approved by the Institutional Animal Care and Use Committee at Beth Israel Deaconess Medical Center. The *Slc25a47* floxed (*Slc25a47*^flox/flox^) mouse was generated by in vitro fertilization of homozygous sperm (UC David) from Slc25a47^tm1a (EUCOMM)Hmgu^ targeting exons 5 and 6 of the *Slc25a47* gene in C57BL/6J background. A floxed LacZ-neomycin cassette on the Tm1a allele was removed using a flippase (FLP)/Frt deletion by breeding *Slc25a47*^flox/flox^ with FLP deleter mice (Jackson Laboratory, Stock No. 009086). *Slc25a47*^flox/flox^ mice were bred with Albumin Cre mice (Jackson Laboratory, Stock No. 003574) to generate liver-specific *Slc25a47* deletion mice (*Slc25a47^Alb-Cre^*). Mice were kept under a 12-h:12-h light–dark cycle at ambient temperature (22 to 23 °C) and had free access to food and water. Mice were maintained on a regular chow diet or fed with a high-fat diet (60% fat, D12492, Research Diets) starting from 6 wk of age for 6 wk. All mice were fasted for 6 h before killing. To acutely deplete *Slc25a47*, we injected 7-wk-old *Slc25a47*^flox/flox^ mice with 1.5 × 10^11^ genome copies of AAV8-TBG-Cre (Addgene, 107787-AAV8) or AAV8-TBG-null (control, Addgene, 105536-AAV8) through tail vein injection.

### Human SNP Analyses.

Data were obtained from the Type 2 Diabetes Knowledge Portal (type2diabetesgenetics.org) and reconstructed. We used the SLC25A47 gene as the primary locus and expanded 5,000 bp proximal and distal to the total gene distance in order to identify regions of interest that may be outside of the coding sequence, i.e., promoters or enhancers.

### ^13^C-Glucose and ^13^C-Lactate Infusion Study.

Jugular vein catheters (Instech Labs) were implanted in the right jugular vein of 10-wk-old mice (*n* = 6 per group) under aseptic conditions. The catheter was connected to a vascular access button (Instech Labs) into which the tracer was infused. After 1 wk of the recovery period, mice were fasted for 6 h, and then infused for 2.5 h with U-^13^C-glucose (0.2 M, CLM-1396), U-^13^C-sodium lactate (0.49 M, CLM-1579), 0.2 M ^13^C-alanine (0.2 M, CLM-2184-H), and U-^13^C-glycerol (0.1M, CLM15101), respectively, at 2 to 3-d interval. The infusion rate was 0.1 µL g^−1^ min^−1^, and mice moved freely in a cage during the intravenous infusions. Blood (~ 10 µL) was collected from the tail into microvettes with coagulating activator (Starstedt Inc, 16.440.100). Blood samples were kept on ice, and serum was separated by centrifugation at 3,000 g for 10 min at 4 °C. 4 µL serum was added to 60 µL ice-cold extraction solvent (methanol: acetonitrile: water at 40:40:20), vortexed vigorously and incubated on ice for at least 5 min. The samples were centrifuged at 16,000 g for 10 min at 4 °C, and the supernatant was transferred to LC–MS tubes for analyses.

### Calculation of Direct Contribution Fraction of Gluconeogenic Substrates to Glucose.

The calculation follows the method as prior reported (50). Briefly, for a metabolite with carbon number C *,* the labeled isotopologue is noted as [M+i] , and its fraction is noted as $L_{\left[M+i\right]}$ , with i being the number of ^13^C atoms in the isotopologue. The overall ^13^C labeling $L_{\mathrm{metabolite}}$ of the metabolite is calculated as the weighted average of atomized labeling of all isotopologues, or mathematically,

$$L_{\mathrm{metabolite}}=\sum_{i=o}^{n}\frac{i}{C}L_{\left[M+i\right]}$$

The normalized labeling $L_{metabolite\leftarrow tracer}$ is defined as the labeling of a metabolite normalized by the labeling of the infused tracer, as

$$L_{metabolite\leftarrow tracer}=\frac{L_{\mathrm{metabolite}}}{L_{\mathrm{tracer}}}$$

As such, the direct contribution of gluconeogenic substrates to glucose production is algebraically calculated by solving the matrix equation

$$\left[\begin{array}{ccc} 1 & L_{gly\leftarrow lac} & L_{ala\leftarrow lac}\\ L_{lac\leftarrow gly} & 1 & L_{ala\leftarrow gly}\\ L_{lac\leftarrow ala} & L_{gly\leftarrow ala} & 1 \end{array}\right]\;\left[\begin{array}{c} f_{\mathrm{lac}}\\ f_{\mathrm{gly}}\\ f_{\mathrm{ala}} \end{array}\right]=\left[\begin{array}{c} L_{glu\leftarrow lac}\\ L_{glu\leftarrow gly}\\ L_{glu\leftarrow ala} \end{array}\right]$$

Specifically, let M be the matrix and f the vector on the left side, and L the vector on the right side. The operation seeks to

$$\begin{array}{c} min\;∥\;M\cdot f-L\;∥,\;subject\;to\;vector\;f\geq vector\;0 \end{array}$$

The equation is solved using the R package *limSolve* (51). The error was estimated using Monte Carlo simulation by running the matrix equation 100 times, each time using randomly sampled $L_{metabolite\leftarrow tracer}$ values drawn from a normal distribution based on the mean and SE of entries in M and f . The calculated f 's were pooled to calculate the error. This scheme was extended to calculate the mutual interconversions among the metabolites. The peak intensity of each measured isotope was corrected by natural abundance. To calculate the fraction of ^13^C-labeled carbon atoms of glucose, pyruvate, lactate, glutamine, and alanine derived from ^13^C-glucose and ^13^C-lactate, percent ^13^C enrichment (%) was first calculated from the data corrected by natural abundance and then normalized based on the serum tracer enrichment.

### ^13^C-Tracers in the Liver Mitochondria.

Fifty microliters isolated mitochondrial suspension was added into 450 uL modified KPBS (136 mM KCL, 10 mM KH_2_PO_4_, 10 mM HEPES, pH 7.25) containing 2 mM U-^13^C pyruvate (Cambridge Isotopes, CLM-2440-0.1) and incubated on ice for 5 min. After incubation, samples were centrifuged at 10,000 g × 30 s, and washed three times by adding 1 mL ice-cold KPBS. Subsequently, the supernatant was removed and 1 mL ice-cold LC/MS 80% methanol was added. To completely extract metabolites from the mitochondria, the sample was homogenized using TissueLyser II (Qiagen, 85300) for 5 min at 30 Hz, followed by centrifugation at 20,000 g for 15 min at 4 °C. The supernatant was kept on dry ice, and the pellet was resuspended with 500 uL 80% LC/MS-grade methanol, vortexed vigorously, and allowed to extract on ice. The samples were then centrifuged at 20,000 g for 10 min at 4 °C. The extraction was vacuum dried using a vacuum concentrator (Eppendorf, concentrator Plus 5305). Dried samples were solubilized in 50 µL LC/MS water. Metabolite analysis was conducted at the BIDMC metabolomics core. The data were normalized by protein concentration.

## Supplementary Material

Appendix 01 (PDF)

## Data Availability

Previously published data were used for this work (36). Human SNP data were obtained from the Type 2 Diabetes Knowledge Portal (type2diabetesgenetics.org). scATAC-seq and ChIP-seq data were obtained from GEO (GSE111586 and GSE90533, respectively). For the analysis of SLC25A47 gene expression in human tissues and single cells of the human liver, the data was obtained from Human Protein Atlas (https://www.proteinatlas.org/ENSG00000140107-SLC25A47/tissue and https://www.proteinatlas.org/ENSG00000140107-SLC25A47/single+cell+type/liver, respectively). The data for mouse Slc25a47 expression in tissues was obtained from GTEx portal (https://www.gtexportal.org/home/gene/SLC25A47).
