# Supplementary material for: The SLC25A47 locus controls gluconeogenesis and energy expenditure
Source: Proc Natl Acad Sci U S A. 2023 Feb 22;120(9):e2216810120. doi: 10.1073/pnas.2216810120 (PMC9992842; doi:10.1073/pnas.2216810120)
Supplement: Supplementary file 1 — Appendix 01 (PDF) [file pnas.2216810120.sapp.pdf]

PNAS

Supporting Information for
The SLC25A47 locus controls gluconeogenesis and energy expenditure

Jin-Seon Yook, Zachary H. Taxin, Bo Yuan, Satoshi Oikawa, Christopher Auger, Beste Mutlu, Pere Puigserver, Sheng Hui, and Shingo Kajimura*

*Corresponding Author: skajimur@bidmc.harvard.edu

This PDF file includes:

Supporting text
SI References
Figures S1 to S6
Tables S1

Supporting Information Text

RNA-Sequencing: Extracted RNA (400ng) from WT or *Slc25A47*$^{Alb\text{-}Cre}$ liver samples were treated with NEBNext rRNA Depletion Kit v2 (E7400X) to remove ribosomal RNA. First stand was synthesized using Thermo Scientific Random Hexamer Primer (SO142) and Maxima Reverse Transcriptase (EP0742). Second strand was synthesized using NEBNext mRNA Second Strand Synthesis Module (E6111L). cDNA was analyzed with Qubit and Agilent BioAnalyzer, and subsequently tagmented and amplified for 12 cycles using Nextera XT DNA Library Preparation Kit (Illumina FC-131). Generated libraries were analyzed with Qubit and Agilent Bioanalyzer, pooled at a final concentration of 1.35 pM, and sequenced on a NextSeq 500. Sequenced reads were demultiplexed and trimmed for adapters using bcl2fastq (v2.20.0). Secondary adapter trimming, NextSeq/Poly(G) tail trimming, and read filtering was performed using fastp (v0.23.2) (1); low quality reads and reads shorter than 24nt after trimming were removed from the read pool. Salmon (v1.6.0) (2) was used to simultaneously map and quantify reads to transcripts in the GENCODE M26 genome annotation of GRCm39/mm39 mouse assembly. Salmon was run using full selective alignment, with sequence-specific and fragment GC-bias correction turned on (seqBias and gcBias options, respectively). Transcript abundances were collated and summarized to gene abundances using the tximport package for R (3). Normalization and differential expression analysis were performed using edgeR (4, 5). For differential gene expression analysis, genes were considered significant if they passed a fold change (FC) cutoff of log2FC < 1 and a false discovery rate (FDR) cutoff of FDR < 0.05.

Transcriptional analysis: scATAC-seq and ChIP-seq data were obtained from GEO (GSE111586 and GSE90533, respectively) and visualized using IGV (Integrative Genomics Viewer). For the analysis of SLC25A47 gene expression in human tissues and single cells of the human liver, the data was obtained from Human Protein Atlas (https://www.proteinatlas.org/ENSG00000140107- SLC25A47/tissue and https://www.proteinatlas.org/ENSG00000140107- SLC25A47/single+cell+type/liver, respectively). The data for mouse Slc25a47 expression in tissues was obtained from GTEx portal (https://www.gtexportal.org/home/gene/SLC25A47). From these data, RNA expression at the tissue and single cell level was reconstructed.

Serum metabolite analysis in serum by LC/MS: Chromatographic separation was achieved using XBridge BEH Amide XP Column (2.5 µm, 2.1 mm × 150 mm) with guard column (2.5 µm, 2.1 mm X 5 mm) (Waters, Milford, MA). The mobile phase A was water: acetonitrile 95:5, and mobile phase B was water: acetonitrile 20:80, both phases containing 10 mM ammonium acetate and 10 mM ammonium hydroxide. The linear elution gradient was: 0 ~ 3 min, 100% B; 3.2 ~ 6.2 min, 90% B; 6.5. ~ 10.5 min, 80% B; 10.7 ~ 13.5 min, 70% B; 13.7 ~ 16 min, 45% B; and 16.5 ~ 22 min, 100% B, with flow rate of 0.3 mL/ min. The autosampler was at 4˚C. The injection volume was 5 µL. Needle wash was applied between samples using methanol: acetonitrile: water at 40: 40: 20. The mass spectrometer used was Q Exactive HF (Thermo Fisher Scientific, San Jose, CA), and scanned from 70 to 1000 *m/z* with switching polarity. The resolution was 120,000. Metabolites were identified based on accurate mass and retention time using an in-house library, and the software used was EI-Maven (Elucidata, Cambridge, MA). ^{13}C-Natural abundance correction was performed in R using package AccuCor (6).

Glucose, insulin, pyruvate, and glycerol tolerance tests: For glucose and insulin tolerance tests, mice were fasted for 6 h from 9:00 to 15:00 prior to glucose (2 g/kg BW) or insulin (0.4 U/kg BW) injection intraperitoneally. For insulin measurement during the glucose tolerance test, blood was collected 15 min after the glucose administration. For pyruvate and glycerol tolerance tests, we fasted mice for 16 h and injected pyruvate (2 g/kg BW) or glycerol (2 g/kg BW) intraperitoneally. Blood glucose levels were measured at the indicated time points using blood glucose test strips (Freestyle Lite).

Oxygen consumption rate: To isolate mitochondria, liver samples were homogenized in 3 mL of MSHE buffer (70 mM sucrose, 210 mM mannitol, 5 mM HEPES, 1 mM EGTA, pH 7.4) containing 0.5% free fatty acid free BSA on ice using a Teflon pestle (Fisher Scientific, FSR3000) by 5 strokes at 1,500 rpm. The homogenate was transferred into a cold 15 ml and centrifuged at 600 g for 10 min at 4 ˚C. The supernatant was then transferred into multiple cold 1.5 mL EP tubes and spun at 1,100 g for 10 min at 4 ˚C. The pellet was resuspended in 500 µL of MSHE and then centrifuged at 8,000 g for 10 min at 4 ˚C. To remove BSA for protein quantification, the pelleted mitochondria were washed once and resuspended in MSHE without BSA. Oxygen consumption rate in liver mitochondria was measured using the Oroboros Oxygraph 2k

(NextGen-O2k, 10101- 01). Briefly, 80 ug of mitochondria was added into the calibrated Oroboros chamber. To obtain state 4 respiration linked to CI, 5 mM pyruvate, 2 mM malate, and 10 mM glutamate were injected. State 3 respiration was measured via the addition of 4 mM ADP. Injection of 10 mM succinate permitted the measurement of CI and CII-driven respiration.

Serum analyses: Blood was coagulated at room temperature for 1 h, and then serum was separated by centrifugation at 2,000 g for 20 min at 4 ˚C and kept at -80 ˚C until further analysis. Total cholesterol (TC, Stanbio), triglyceride (TG, Thermos Fisher), insulin (Millipore Sigma), FGF21 (R&D system), AST and ALT (Abcam), and albumin (Stanbio) were analyzed in the serum according to the manufacturer's instruction. Glycogen measurement: Glycogen content was determined using a commercially available kit (Abcam). Briefly, 10 mg of liver tissue was homogenized in cold distilled water using two stainless steel beads (TissueLyser II for 1 min at 40 Hz). The samples were then boiled at 95 ˚C for 10 mins to inactivate enzymes, and insoluble material was removed after centrifugation at 18,000 g at 4 ˚C for 10 mins. The supernatant was quantified by colorimetric assays, and glucose level was subtracted from the glycogen levels.

Whole-body energy expenditure analyses: The metabolic variables, including oxygen consumption rate (VO_2), carbon dioxide release rate (VCO_2), energy expenditure (EE), food intake, and locomotor activity (beam break counts), were measured using the Promethion Metabolic Cage System (Sable Systems) at 23 ˚C at 1 week of a high-fat diet, and at 30 ˚C at 3 weeks of a high-fat diet. Body Composition (lean mass and fat mass) was measured using Analyzer EchoMRI (Echo Medical Systems). As for examining metabolic changes after CL-316,243 injection, mice on a regular chow diet were housed at 30˚C for one week prior to CL injection to acclimate to thermoneutrality. The metabolic variables were measured using the Promethion Metabolic Cage System at 30 ˚C before and after CL-316,243 (0.5mg/kg weight *i.p.*) injection.

qRT-PCR: Total RNA was isolated from the liver by using TRIzol Reagent (Invitrogen) and further purified using Zymo direct-zol RNA prep (Zymo research) according to the manufacturer's protocol. Genomic DNA was removed using DNAse during RNA purification, and cDNA was synthesized using iScript™ Reverse Transcription Supermix (Biorad) following the provided protocol. RT-PCR was performed on a QuantStudio™ 6 (Life technologies) using iTaq™ Universal SYBR® Green Supermix (Biorad). Relative mRNA levels were calculated based on the $2^{-\Delta\Delta CT}$ method with normalization of the raw data to 18s. Primer sequences are available in **Supplementary Table 1**.

Histology: Liver samples were fixed in 4% paraformaldehyde (PFA) at 4 ˚C overnight, followed by dehydration in 70% ethanol. After dehydration, the samples were processed for paraffin embedding and cross-sectioned at 5 μm thickness. Sections were subjected to hematoxylin and eosin (H&E) staining or picro-sirius red staining according to the standard protocol. Images were acquired using the Zeiss AxioImager M1 (Carl Zeiss).

Statistical analyses: Statistical analyses were performed using GraphPad Prism 9.4.1 (GraphPad Software) and R studio. All data were represented as mean ± SEM unless otherwise specified. Unpaired Student's t-test and Mann-Whitney test were used for two-group comparisons. Two-way repeated-measures ANOVA followed by Fisher's LSD test was applied to determine the statistical differences in body weight change, whole-body energy expenditure results, glucose tolerance test, insulin tolerance test, and pyruvate tolerance test between genotypes. Testing the direct association between two variables was performed using simple linear regression. The statistical parameters and mice numbers used per experiment are clarified in the figure legends. $p < 0.05$ was considered significant in all the experiments.

SI References

1. S. Chen, Y. Zhou, Y. Chen, J. Gu, fastp: an ultra-fast all-in-one FASTQ preprocessor. *Bioinformatics* **34**, i884-i890 (2018).
2. R. Patro, G. Duggal, M. I. Love, R. A. Irizarry, C. Kingsford, Salmon provides fast and bias-aware quantification of transcript expression. *Nature methods* **14**, 417-419 (2017).
3. C. Soneson, M. Love, M. Robinson Differential analyses for RNA-seq: transcript-level estimates improve gene-level inferences. *F1000Res* **4**: 1521 (2015).
4. M. D. Robinson, D. J. McCarthy, G. K. Smyth, edgeR: a Bioconductor package for differential expression analysis of digital gene expression data. *Bioinformatics* **26**, 139- 140 (2010).
5. Y. Chen, A. T. Lun, G. K. Smyth, From reads to genes to pathways: differential expression analysis of RNA-Seq experiments using Rsubread and the edgeR quasi-likelihood pipeline. *F1000Research* **5** (2016).
6. X. Su, W. Lu, J. D. Rabinowitz, Metabolite spectral accuracy on orbitraps. *Analytical chemistry* **89**, 5940-5948 (2017)

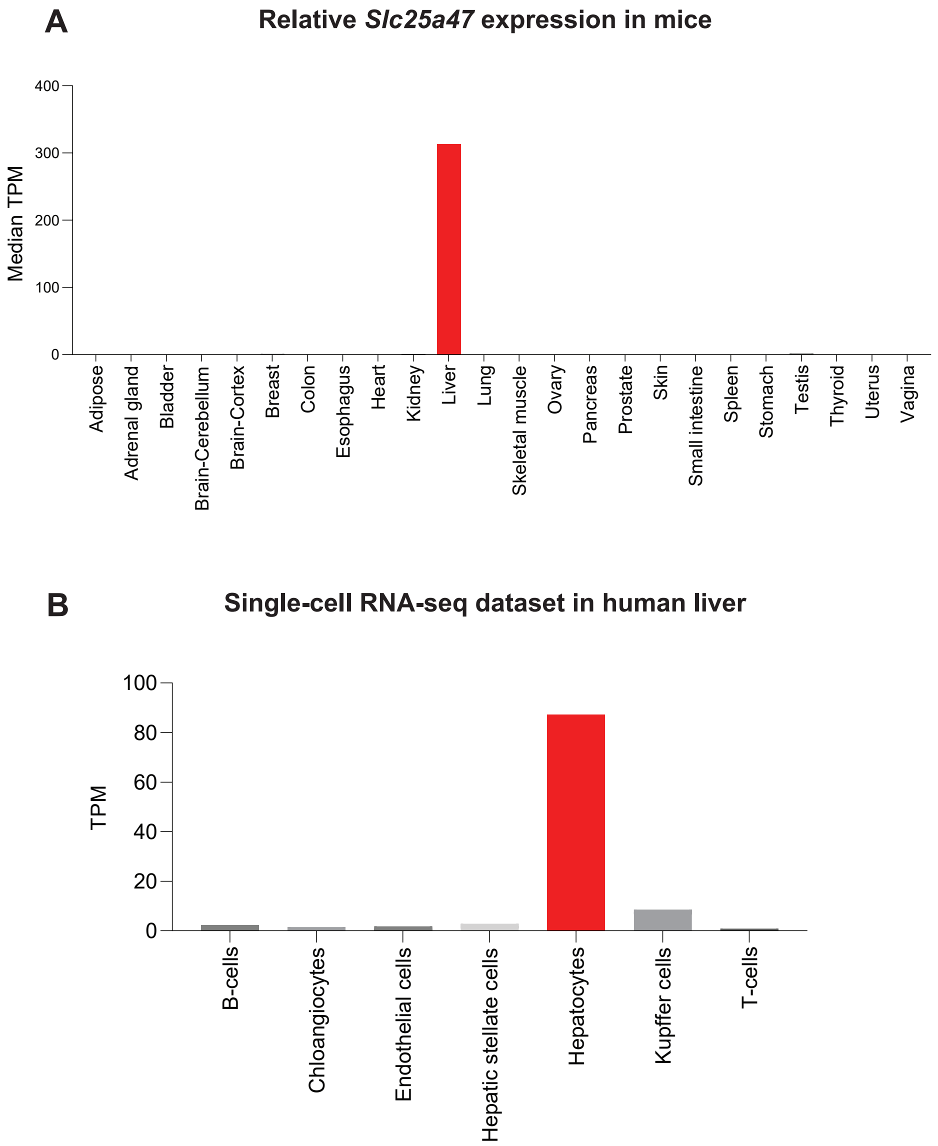

Supplementary Figure S1. SLC25A47 is a liver-specific mitochondrial carrier in mice.

A. Relative mRNA levels (TPM) in indicated tissues of mice. The data obtained from GTEx portal (https://www.gtexportal.org/home/gene/SLC25A47) were analyzed.

B. Relative mRNA levels (TPM) in indicated cell types in the human liver. The data obtained from https://www.proteinatlas.org/ENSG00000140107-SLC25A47/single+cell+type/liver were analyzed.

A **Log2 fold change of *Slc25a47* neighboring genes**

Neighborhood	Gene	mean log2 CPM	log2 fold change	FDR
Downstream	*Wdr25*	1.91	-0.33	0.26
	Begain	not expressed in the liver (TPM <1)		
	Dlk1	not expressed in the liver (TPM <1)		
	Meg3	2.53	0.23	0.82
Upstream	*Slc25a29*	2.14	-0.07	0.86
	Yy1	6.36	0.03	0.87
	Degs2	not expressed in the liver (TPM <1)		

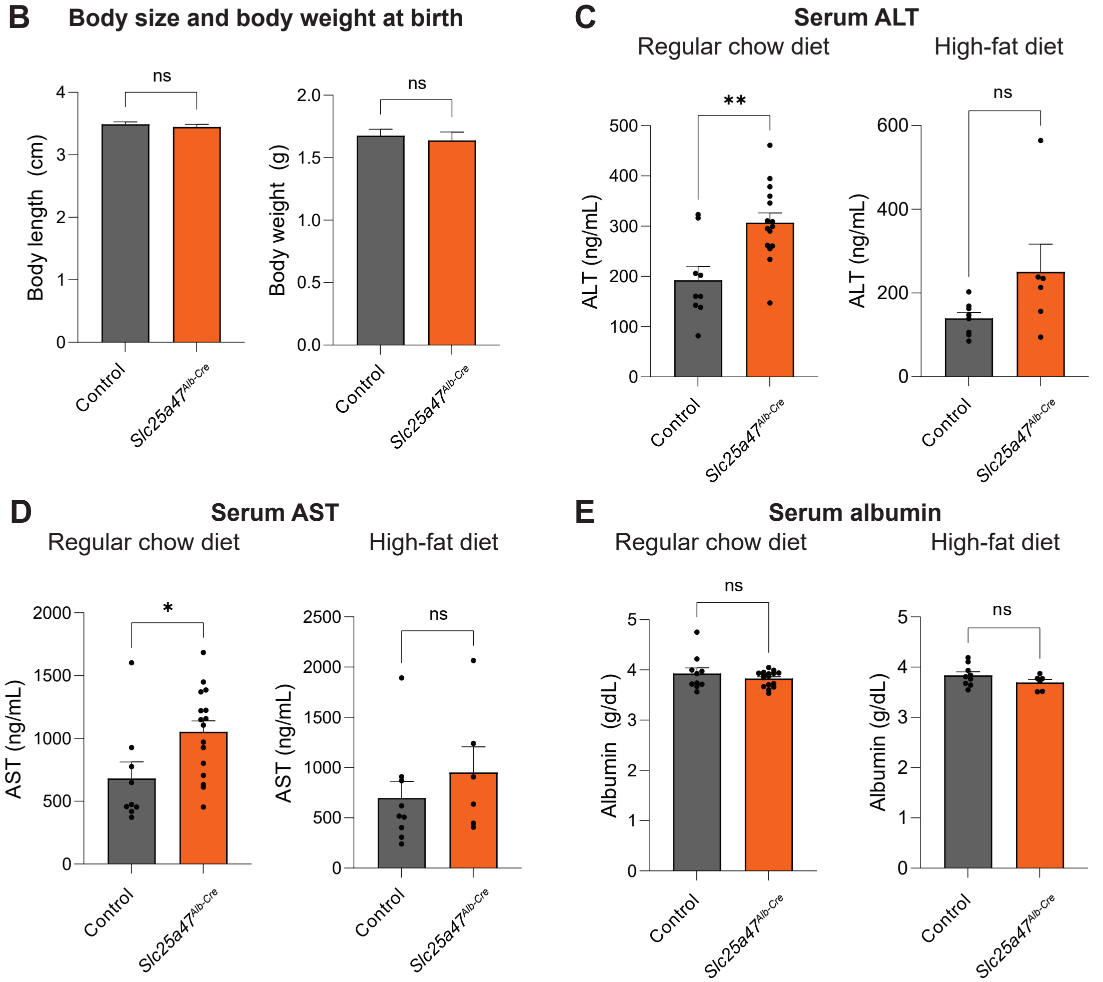

Supplementary Figure S2. Characterization of *Slc25a47*$^{Alb\text{-}Cre}$ mice.

A. The expression of indicated *Slc25a47* neighboring genes in the liver of *Slc25a47*$^{Alb\text{-}Cre}$ mice and controls. n = 7 for *Slc25a47*$^{Alb\text{-}Cre}$, n = 6 for controls. FDR adjusted *P*-value was calculated using Benjamini-Hochberg method.

B. Body length and body weight in *Slc25a47*$^{Alb\text{-}Cre}$ mice and littermate controls in newborn. n = 10 for *Slc25a47*$^{Alb\text{-}Cre}$, n = 18 for controls. Data are mean ± SEM.; ns, not significant, by two-tailed unpaired Student's *t*-test.

C. Serum ALT levels of *Slc25a47*$^{Alb\text{-}Cre}$ and littermate control mice on a regular chow diet and high-fat diet. Regular chow diet: n = 16 for *Slc25a47*$^{Alb\text{-}Cre}$ mice, n = 10 for controls. High-fat diet: n = 6 for *Slc25a47*$^{Alb\text{-}Cre}$, n = 9 for controls. * $p < 0.05$, ** $p < 0.01$, ns, not significant, by two-tailed unpaired Student's *t*-test.

D. Serum AST levels of mice in (C).

E. Serum albumin levels of mice in (C).

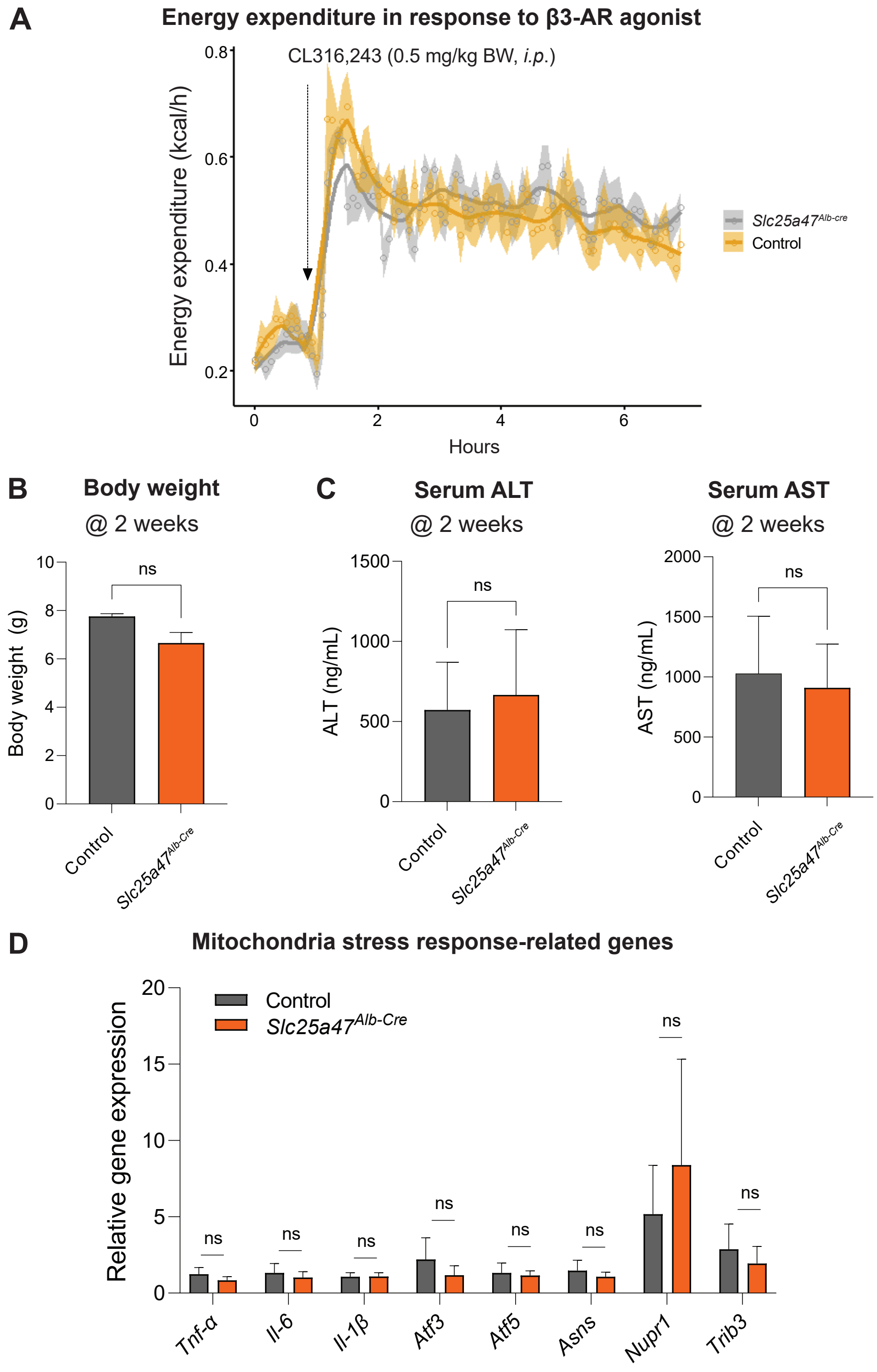

Supplementary Figure S3. Brown adipose tissue thermogenesis and liver function of *Slc25a47*$^{Alb\text{-}Cre}$ mice.

- **A.** Energy expenditure (kcal/h) of *Slc25a47*$^{Alb\text{-}Cre}$ mice and littermate controls on a regular chow diet. Mice kept at 30˚C received *i.p.* injection of CL316,243 at 0.5 mg kg^{-1} at the indicated time point (arrow). n = 8 for both groups, biologically independent mice. Data are mean ± SEM.; *P*-value was determined by two-way repeated-measures ANOVA.
- **B.** Body weight of mice at 2 weeks age. n = 6 for *Slc25a47*$^{Alb\text{-}Cre}$, n = 4 for controls. ns, not significant, by two-tailed unpaired Student's *t*-test.
- **C.** Serum ALT (left) and AST (right) levels of mice at 2 weeks of age in (B).

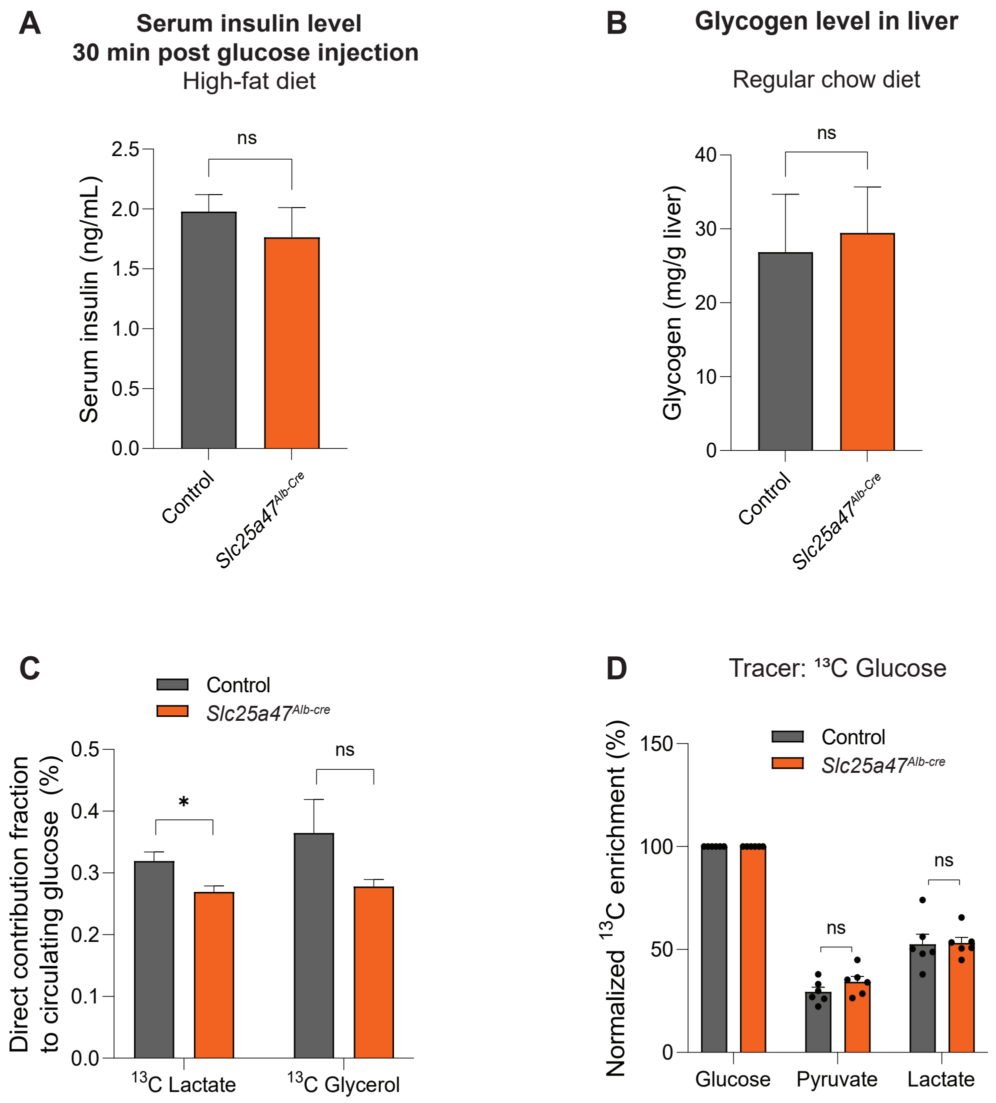

Supplementary Figure S4. Glucose metabolism of *Slc25a47$^{Alb\text{-}Cre}$* mice.

A. Glucose-stimulated serum insulin levels at 30 minutes after glucose injection in mice on a high-fat diet. n = 10 for *Slc25a47$^{Alb\text{-}Cre}$*, n = 5 for controls. Data are mean ± SEM.; ns, not significant, by two-tailed unpaired Student's t-test.

B. Glycogen levels in the liver of mice at 12 weeks of age on a regular chow diet. n = 16 for *Slc25a47$^{Alb\text{-}Cre}$*, n = 9 for controls.

C. Direct contribution of ^{13}C-labeled glucose and glycerol to circulating glucose. n = 6 for *Slc25a47$^{Alb\text{-}Cre}$*, n = 6 for controls. * $p < 0.05$, ns, not significant, by unpaired Student's t-test.

D. ^{13}C-enrichment (%) of labeled glucose to circulating levels of indicated metabolites in (C). ns, not significant, by unpaired Student's t-test.

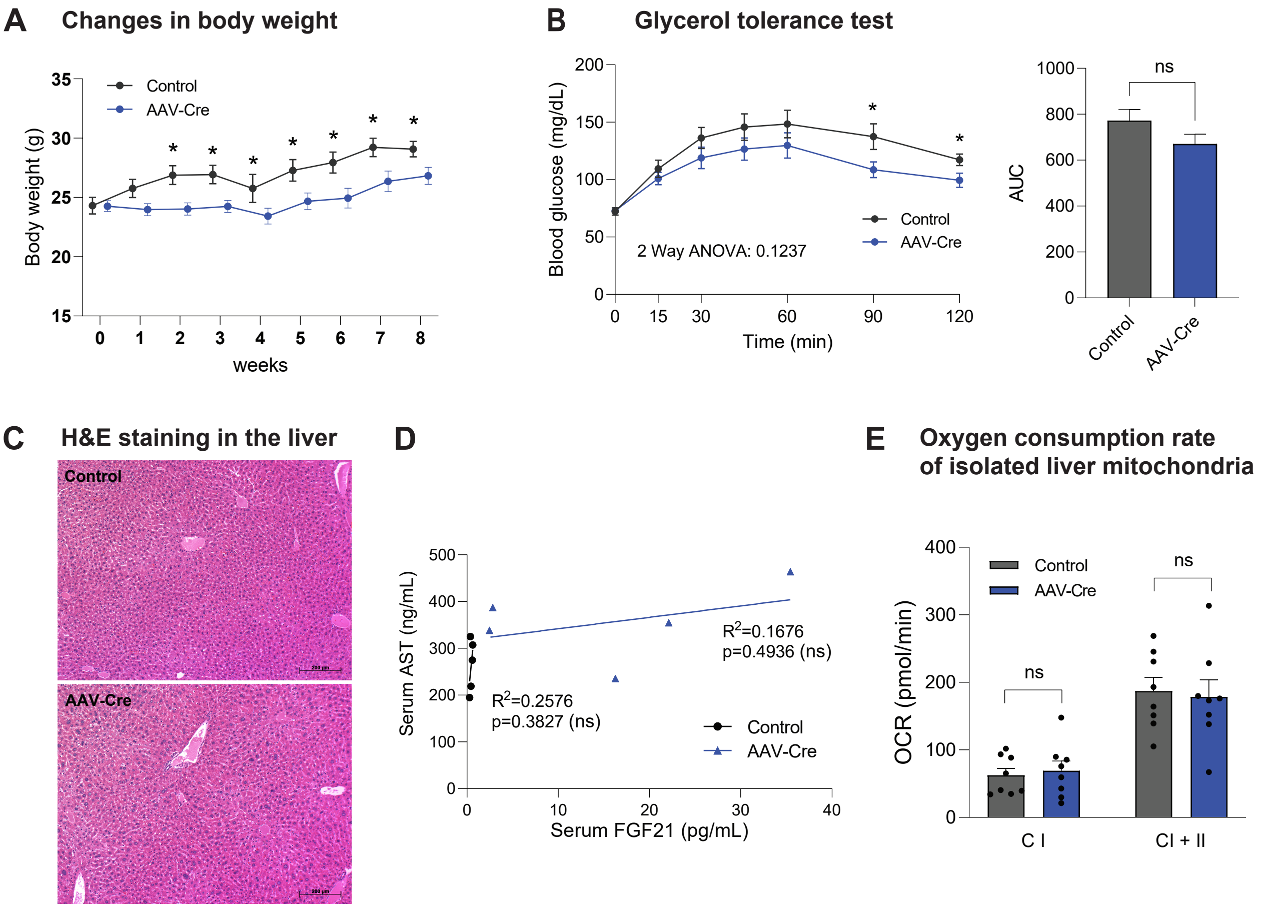

Supplementary Figure S5. Metabolic changes in response to acute SLC25A47 depletion in adult mice.

A. Changes in body weight of *Slc25a47*$^{flox/flox}$ mice that received AAV-Cre or AAV-null (control) at indicated time points. Mice were on a regular chow diet. *n* = 10 for controls, *n* = 13 for AAV-Cre, biologically independent mice. Data are mean ± SEM.; *P*-value was determined by unpaired Student's *t*-test.

B. Glycerol tolerance test in *Slc25a47*$^{flox/flox}$ mice at 7 weeks after receiving AAV-Cre or AAV-null (control). After 16 hours of fasting, mice received *i.p.* injection of glycerol at 2 g kg^{-1} body-weight. *n* = 11 for controls, *n* = 14 for AAV-Cre, biologically independent mice. *P*-value was determined by two-way repeated-measures ANOVA followed by Fisher's LSD test. Right: Area under the curve (AUC) of the data was calculated by Graphpad software. ns, not significant, by two-tailed unpaired Student's *t*-test.

C. Representative image of hematoxylin and eosin (H&E) staining in the liver of *Slc25a47*$^{flox/flox}$ mice at 2 weeks after receiving AAV-Cre or AAV-null (control). Scale = 200 μm.

D. Correlation between serum FGF21 levels and serum AST levels in *Slc25a47*$^{flox/flox}$ mice at 2 weeks after receiving AAV-Cre or AAV-null (control). *n* = 5 for controls, *n* = 5 for AAV-Cre.

E. Oxygen consumption rate (OCR) of isolated mitochondria from the liver of *Slc25a47*$^{flox/flox}$ mice that received AAV-Cre or AAV-null (control). Mitochondrial Complex I (CI) activity was measured by adding glutamate (10 mM), pyruvate (5 mM), and malate (2 mM). Succinate (10 mM) was then added to measure the respiration of complexes I and II. *n* = 8 for controls, *n* = 8 for AAV-Cre. ns, not significant, by two-tailed unpaired Student's *t*-test.

Metabolites in liver mitochondria

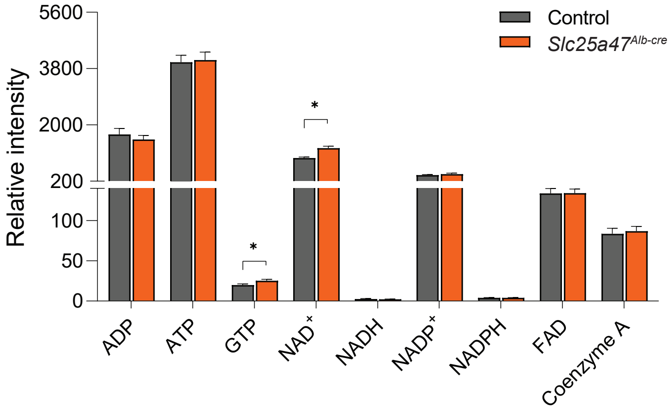

Supplementary Figure S6. Mitochondrial metabolomics of the liver.

Relative levels of indicated metabolites in the liver mitochondria from *Slc25a47*$^{Alb\text{-}Cre}$ mice and littermate controls in Fig. 5C. Mice at 7 weeks of age fasted for 6 hours. n = 14 for *Slc25a47*$^{Alb\text{-}Cre}$, n = 14 for controls. Data were normalized to mitochondrial protein levels and shown as mean ± SEM.; P-value was determined by two-tailed unpaired Student's t-test.

Supplemental Table 1. List of mouse primer sequences for qPCR

Gene	Forward	Reverse
Slc25a47	TCCAGCCACCAACACACCTA	GAGGGTCACTCCTTTGCCAC
Fgf21	GTGTCAAAGCCTCTAGGTTTCTT	GGTACACATTGTAACCGTCCTC
Tnfα	ATGGCCTCCCTCTCATCAGT	TTTGCTACGACGTGGGCTAC
Il1β	ATGCCACCTTTTGACAGTGAT	AGCCCTTCATCTTTTGGGGT
Il6	CCCCAATTTCCAATGCTCTCC	GGATGGTCTTGGTCCTTAGCC
Trib3	CAGGAAGAAACCGTTGGAGTT	CCAAAAGGATATAAGGCCCCAGT
Nupr1	ACCCTTCCCAGCAACCTCTAA	TCTTGGTCCGACCTTTCCGA
Ddit3	CTGGAAGCCTGGTATGAGGAT	CAGGGTCAAGAGTAGTGAAGGT
Atf3	GAGGATTTTGCTAACCTGACACC	TTGACGGTAACTGACTCCAGC
Atf4	AAGGAGGAAGACACTCCCTCT	CAGGTGGGTCATAAGGTTTGG
Aft5	TGGGCTGGCTCGTAGACTAT	AGTCCACCCGCTCAGTCAT
Asns	CACAAGGCGCTACAGCAAC	CCAGCATACAGATGGTTTTCTCG
Col1a1	TGCTAACGTGGTTCGTGACCGT	ACATCTTGAGGTCGCGGCATGT
Col1a2	TTGCTGAGGGCAACAGCAGGTT	AATGTCAAGGAACGGCAGGCGA
Timp1	CCTTGCAAACTGGAGAGTGACA	AGGCAAAGTGATCGCTCTGGT
α-sma	GTCCCAGACATCAGGGAGTAA	TCGGATACTTCAGCGTCAGGA
Tgfβ	TTGCCCTCTACAACCAACACAA	GGCTTGCGACCCACGTAGTA
18s	AGTCCCTGCCCTTTGTACACA	CGATCCGAGGGCCTCACT
